# Comparing the Diagnostic Performance of Contrast-Enhanced Mammography and Breast MRI: a Systematic Review and Meta-Analysis

**DOI:** 10.7150/jca.79747

**Published:** 2023-01-01

**Authors:** Lidewij M.F.H. Neeter, M.M. Quirien. Robbe, Thiemo J.A. van Nijnatten, Maxine S. Jochelson, H.P.J. Raat, Joachim E. Wildberger, Marjolein L. Smidt, Patty J. Nelemans, Marc B.I. Lobbes

**Affiliations:** 1 GROW School for Oncology and Reproduction, Maastricht University, Universiteitssingel 40, 6229 ER Maastricht, the Netherlands.; 2 Department of Radiology and Nuclear Medicine, Maastricht University Medical Center+, P. Debyelaan 25, 6229 HX Maastricht, the Netherlands.; 3 Department of Radiology, Memorial Sloan Kettering Cancer Center, 1275 York Avenue, New York, NY 10065, USA.; 4 Department of Medical Imaging, Laurentius hospital, Mgr. Driessenstrtaat 6, 6040AX Roermond, the Netherlands.; 5 Department of Surgery, Maastricht University Medical Center+, P. Debyelaan 25, 6229 HX Maastricht, the Netherlands.; 6 Department of Epidemiology, Maastricht University, P. Debyelaan 1, 6229 HA Maastricht, the Netherlands.; 7 Department of Medical Imaging, Zuyderland Medical Center, Dr. H. van der Hoffplein 1, 6162 BG Sittard-Geleen, the Netherlands.

**Keywords:** contrast-enhanced mammography, breast MRI, diagnostic accuracy

## Abstract

**Background:** To provide a systematic review and meta-analysis that evaluates the diagnostic accuracy of contrast-enhanced mammography (CEM) compared to standard contrast-enhanced breast magnetic resonance imaging (breast MRI). Like breast MRI, CEM enables tumour visualization by contrast accumulation. CEM seems to be a viable substitute for breast MRI.

**Methods:** This systematic search assessed the diagnostic accuracy of these techniques in women with suspicious breast lesions on prior imaging or physical examination, who have undergone both breast MRI and CEM. CEM had to be performed on a commercially available system. The MRI sequence parameters had to be described sufficiently to ensure that standard breast MRI sequence protocols were used. Pooled values of sensitivity, specificity, positive likelihood ratio, negative likelihood ratio, and diagnostic odds ratio (DOR), were estimated using bivariate mixed-effects logistic regression modeling. Hierarchical summary receiver operating characteristic curves for CEM and breast MRI were also constructed.

**Results:** Six studies (607 patients with 775 lesions) met the predefined inclusion criteria. Pooled sensitivity was 96% for CEM and 97% for breast MRI. Pooled specificity was 77% for both modalities. DOR was 79.5 for CEM and 122.9 for breast MRI. Between-study heterogeneity expressed as the *I^2^*-index was substantial with values over 80%.

**Conclusion:** Pooled sensitivity was high for both CEM and breast MRI, with moderate specificity. The pooled DOR estimates, however, indicate higher overall diagnostic performance of breast MRI compared to CEM. Nonetheless, current scientific evidence is too limited to prematurely discard CEM as an alternative for breast MRI.

## Introduction

Breast cancer is currently the most common cancer in women, with 2.3 million newly diagnosed women worldwide in 2020 1,2. (Standard) contrast-enhanced breast magnetic resonance imaging (breast MRI) is considered the best imaging modality for breast cancer detection and evaluation of its extent 3. However, it is associated with high costs, long acquisition and reading times, and limited availability in some regions 3,4. It is contraindicated in patients with claustrophobia, some metal implants or foreign objects in their body, and known hypersensitivity reactions to Gadolinium-based contrast agents 5. Therefore, alternative methods of evaluation must be considered. Hence, breast MRI is not a primary imaging modality in breast imaging, but is only indicated for very specific patient populations 6.

Since its introduction in 2011, the use of contrast-enhanced mammography (CEM) has been steadily increasing in both research and clinical settings 7-10. A CEM examination, in which an iodinated contrast agent is administered followed by the acquisition of a dual-energy mammography, uses the same physiological contrast enhancement principle as breast MRI 8. Reading CEM examinations utilizes the fifth edition of the American College of Radiology Breast Imaging Reporting and Data System (BI-RADS) lexicon for mammography, including the supplemental chapter on CEM, combined with that for MRI without the contrast kinetics evaluation and with some descriptors specific for CEM 11,12. Also, CEM seems to be easy to learn 13-16. The costs of a CEM exam are significantly lower than that of breast MRI and patients tend to prefer CEM over breast MRI 4,17,18. Consequently, CEM is becoming an attractive alternative for breast MRI 19.

Although several systematic reviews and meta-analyses assessing the diagnostic performance of CEM have been published before 20-24, most of them compared CEM with full-field digital mammography. Only two meta-analyses compared the diagnostic accuracy of CEM to that of breast MRI, showing conflicting results 23,24. Both reviews used criteria for eligibility of studies, which may have introduced bias of the results. We therefore conducted a systematic review and meta-analysis, applying inclusion criteria that better correspond with everyday clinical practice, to compare the diagnostic accuracy of CEM and breast MRI.

## Methods

This systematic review followed the checklist of Preferred Reporting Items for a Systematic Review and Meta-analysis of Diagnostic Test Accuracy (PRISMA-DTA) 25.

### Search strategy

A systematic literature search of the electronic databases PubMed (Central) and Embase was conducted to identify all published studies on CEM and breast MRI that reported on accuracy in the diagnostic setting. A combination of MeSH and EMTREE terms were used: “breast malignancy”, “contrast-enhanced mammography”, and “magnetic resonance imaging”. To define breast malignancy, the following keywords were used: “breast tumour”, “breast neoplasms”, “carcinoma”, “malignant neoplasms”, and “cancer”. In this primary search, studies published before May 2022 were identified and no language or publication restrictions were applied, such as publication language and conference abstracts or proceedings. Supplemental to the search, the reference lists of retrieved articles were reviewed for other potentially relevant studies. All the retrieved references were entered into a bibliography management software (Covidence systematic review software, Veritas Health Innovation, Melbourne, Australia) to facilitate the search for duplicate references and assist in our systematic approach.

### Study eligibility

First, duplicate records were removed from the list of studies retrieved from the database search. All titles and abstracts of the remaining studies were independently screened by two researchers (LN and MR) to select studies that addressed the comparative value of CEM and breast MRI for diagnostic accuracy. Reviews, technical reports, letter to editors, comments to published studies, case reports, conference abstracts or proceedings, and publications in languages other than English were not considered eligible. After a first screening process, the full-text of the remaining studies was independently assessed for eligibility. Discrepancies between the two researchers were discussed and consensus was given by a third expert reviewer and radiologist (ML) when thought necessary.

To be eligible for inclusion in the review studies had to meet several inclusion criteria. Patients should have received both CEM and breast MRI for the evaluation of breast lesions. Absolute numbers of true positive (TP), true negative (TN), false positive (FP), and false negative (FN) lesions could be derived from the publication. CEM had to be performed on a commercially available system, not on a prototype system, and using a generally accepted image acquisition protocol. MRI sequence parameters had to be described sufficiently to ensure that standard contrast-enhanced breast MRI sequences were used. The study population had to consist of women with suspicious breast lesions on prior imaging (e.g., full-field digital mammography, ultrasound) or clinical examination. Studies that included only patients with histologically verified breast cancer were excluded, because such studies cannot provide information on the ability of CEM and breast MRI to distinguish between benign and malignant lesions. The estimates of sensitivity and specificity from studies, in which all patients had an already diagnosed index breast tumour, relate to the ability of both modalities to identify additional lesions, which corresponds with a different research question.

### Data extraction

The two reviewers independently extracted the data from the eligible studies following a pre-defined extraction format. Variables that were extracted from all studies were: author and publication information, study population characteristics, CEM system characteristics and imaging protocol, MRI system characteristics and imaging sequences, and information on contrast administration for both CEM and breast MRI.

The QUADAS-2 score, the outcome of a tool for the quality assessment of diagnostic accuracy studies (QUADAS), was used to identify risk of bias and applicability concerns in the included studies 26.

### Statistical analysis

For the comparison of pooled diagnostic parameters between CEM and breast MRI for diagnostic work-up, the bivariate model and hierarchical summary receiving operating characteristic (sROC) model were used 27. Pairs of sensitivity and specificity are jointly analyzed, incorporating any correlation that might exist between these two measures using a random effects approach 28. The bivariate model includes five parameters and for fitting of the model a minimum of 4 studies is required 27. sROC curves with the prediction region, summary point and the confidence region were constructed to visualize the trade-off between sensitivity and specificity 27,29.

Statistical analyses were performed using STATA/SE 14.1 (StataCorp LLC, College Station, TX, USA). The *metandi* command in STATA was used, which provides summary estimates of sensitivity and specificity, positive and negative likelihood ratios and diagnostic odds ratios (DOR) 29. The DOR is the odds of a positive test result on imaging in a case with breast cancer divided by the odds of a negative test result on imaging in a case without breast cancer. The *midas* command in STATA was used to construct Forest plots to give an overview of sensitivity and specificity with 95% confidence intervals of individual studies 30. Between-study heterogeneity was expressed as the *I^2^*-index. This statistic quantifies the percentage of total variation across studies that is due to heterogeneity rather than chance 31.

## Results

### Eligible and extracted studies

**Figure 1:**
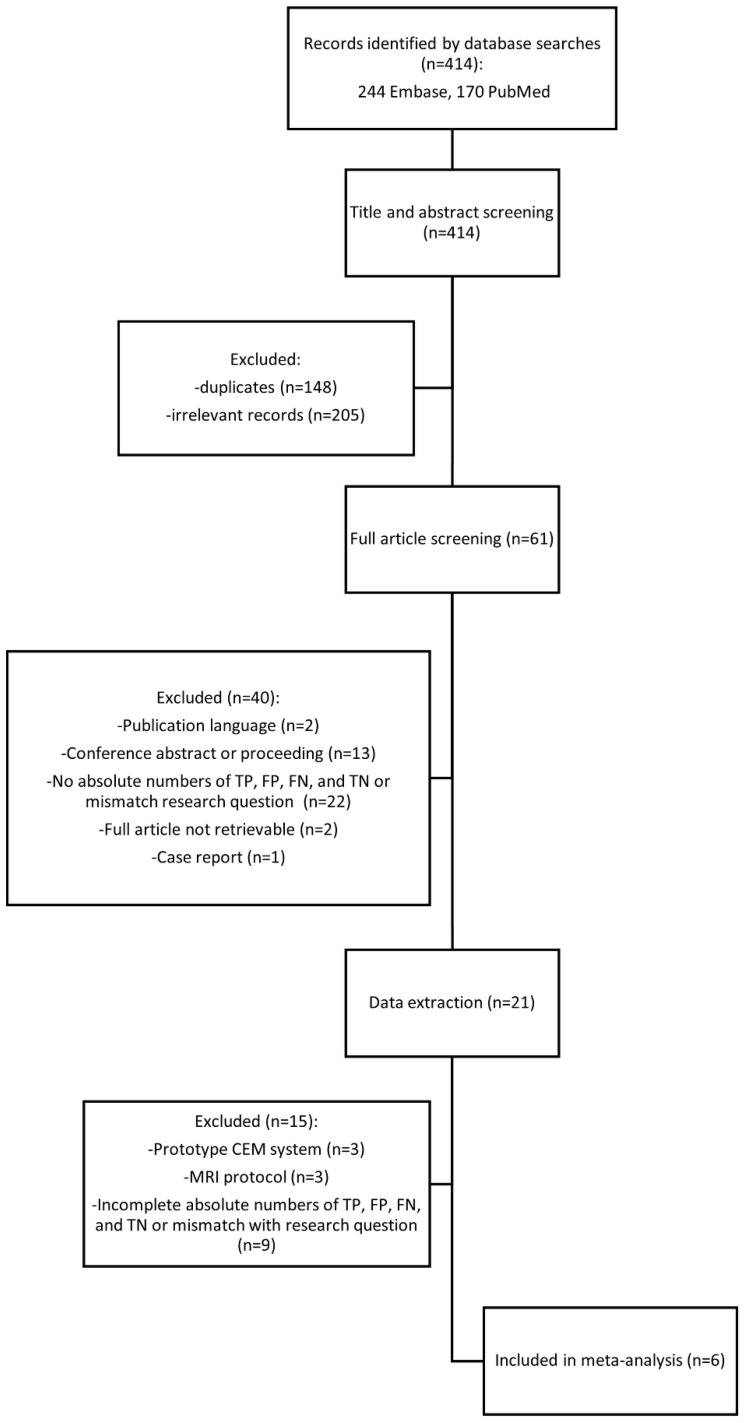
Flowchart of systematic search.

The literature search in Embase and PubMed (Central) databases resulted in the identification of 244 and 170 studies, respectively. After exclusion of 148 duplicate studies, screening of the title and abstract of the remaining 266 studies led to exclusion of another 205 studies. The full text of the 61 remaining studies was retrieved and read, and 21 studies remained for data extraction. During data extraction, three studies were excluded because of lack of sufficient details on MRI sequence parameters. Three other studies were excluded because CEM was performed on a prototype system. Nine studies were excluded because absolute numbers of of true positive (TP), true negative (TN), false positive (FP), and false negative (FN) lesions could not be derived or the study population included only women with histologically verified breast cancer. A list of the excluded publications is given in Supplementary materials 1. In the end, a total of six studies were included in the meta-analysis 32-37. The flow chart in Figure 1 shows the selection of the studies included for review and reasons for exclusion.

### Quality assessment of publications

The QUADAS-2 outcomes are listed in Table 1. Two studies showed low risks of bias and low concern on applicability for all domains 33,36. The four other studies scored high for at least one domain in risk of bias and corresponding domain of concern on applicability 32,34,35,37. A patient population with only BI-RADS 4 lesions resulted in a high score for risk of bias and applicability concerns in the patient selection domain 37. High risk of bias and applicability concern in the index test and reference test domains was assigned in case of unknown reader experience or unknown blinding for the final diagnosis. One study specifically mentioned that the readers had no experience in assessing CEM exams 35. Unknown or no experience in assessing CEM or breast MRI exams might affect the diagnostic accuracy. Not mentioning the time interval between the CEM exam and breast MRI led to a high score of risk of bias in the flow and timing domain 34. With a larger time interval between the two imaging modalities, the tumour could have evolved, probably leading to better visibility, thus better sensitivity for the latter imaging exam.

### Comparison of value of CEM and breast MRI for diagnostic work-up

The included six studies comprised 775 lesions in 607 patients with suspicious breast lesions on prior imaging or clinical examinations, of which 512 lesions were malignant 32-37. One study was conducted on a Hologic system, the remaining studies on GE Healthcare systems. The CEM and breast MRI findings were matched with true disease state using cytological/histopathological results of all lesions, except in two studies. In these two studies lesions assessed as BI-RADS ≤2 on both CEM and breast MRI were considered true negative 35 or the lesions assessed as BI-RADS 2 were closely monitored for one year 32. The same BI-RADS cut-off value was used in the six studies to define the absolute numbers of TP, FP, FN, TN. BI-RADS scores 0-3 were considered as negative cases on imaging and BI-RADS 4-5 as positive cases 32-37. A comprehensive overview of the study characteristics is given in Table 2.

**Figure 2:**
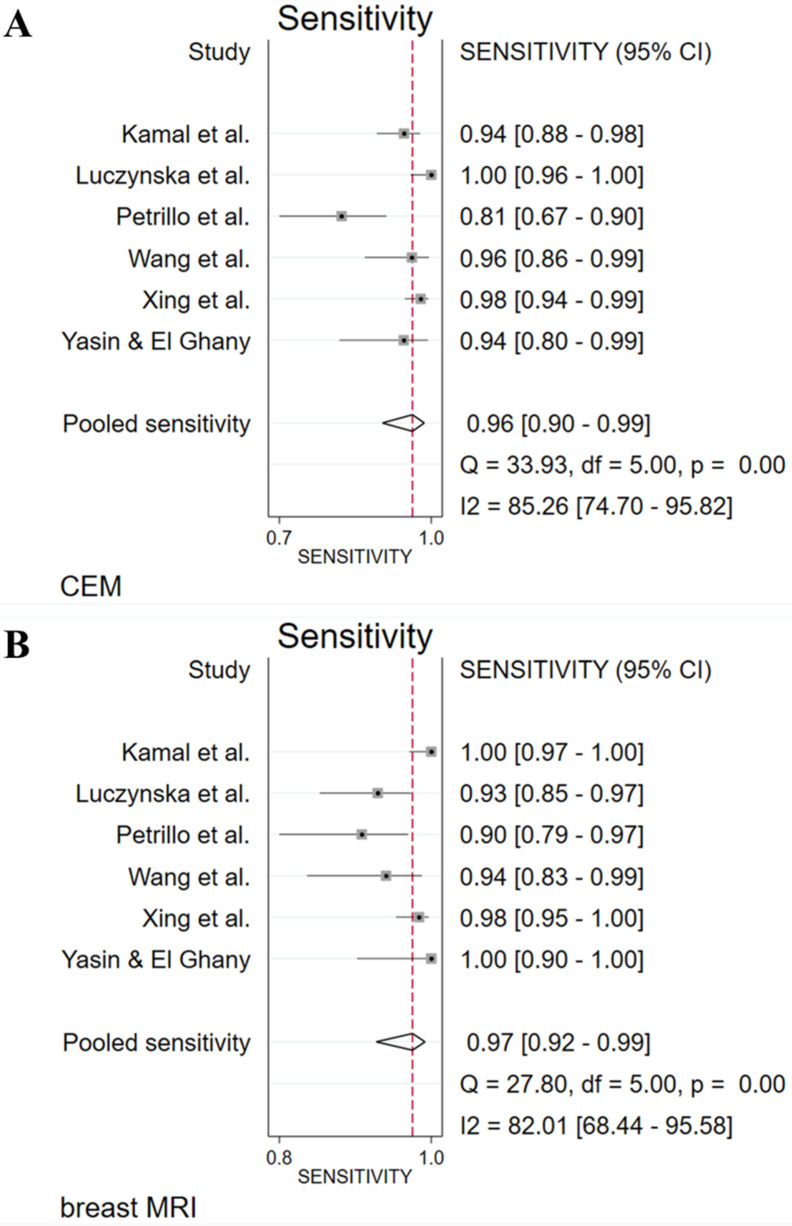
Forest plots of sensitivity for CEM (A) and breast MRI (B) with pooled values, and the I^2^ values.

**Figure 3:**
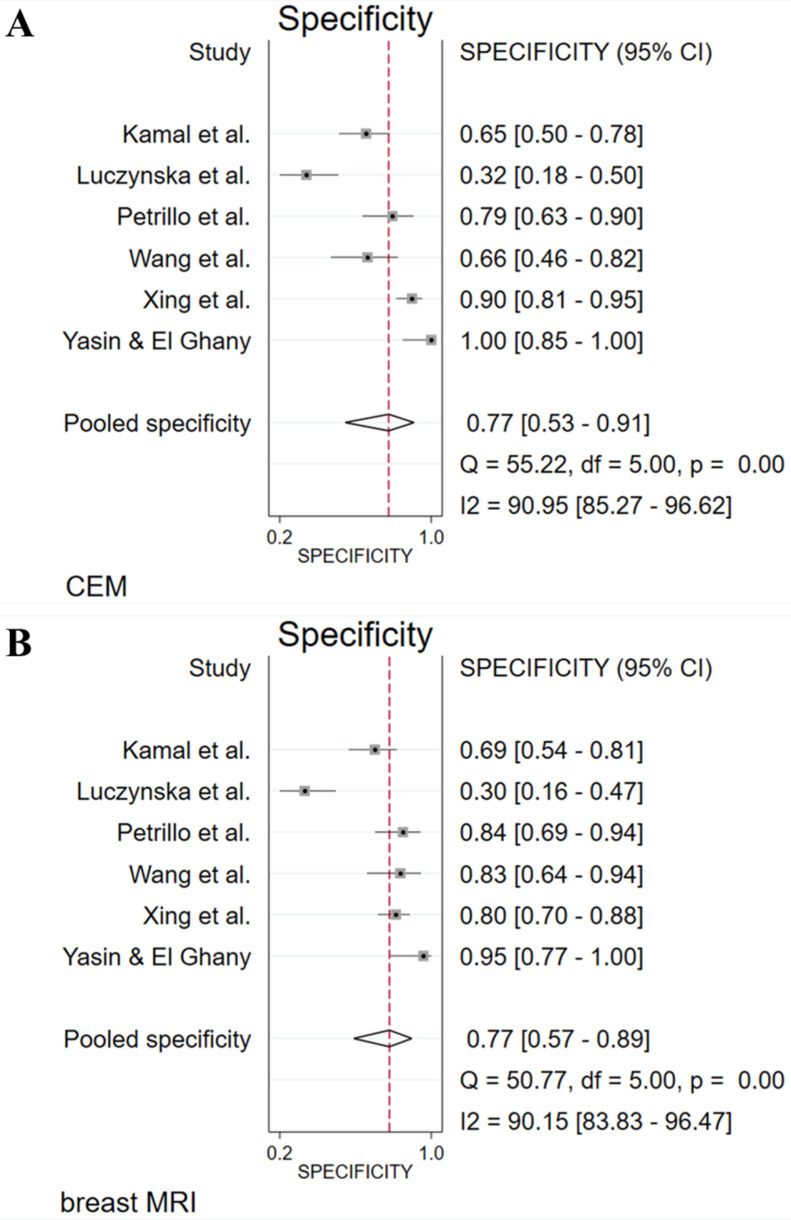
Forest plots of specificity for CEM (A) and breast MRI (B) with pooled values, and the I^2^ values.

**Figure 4:**
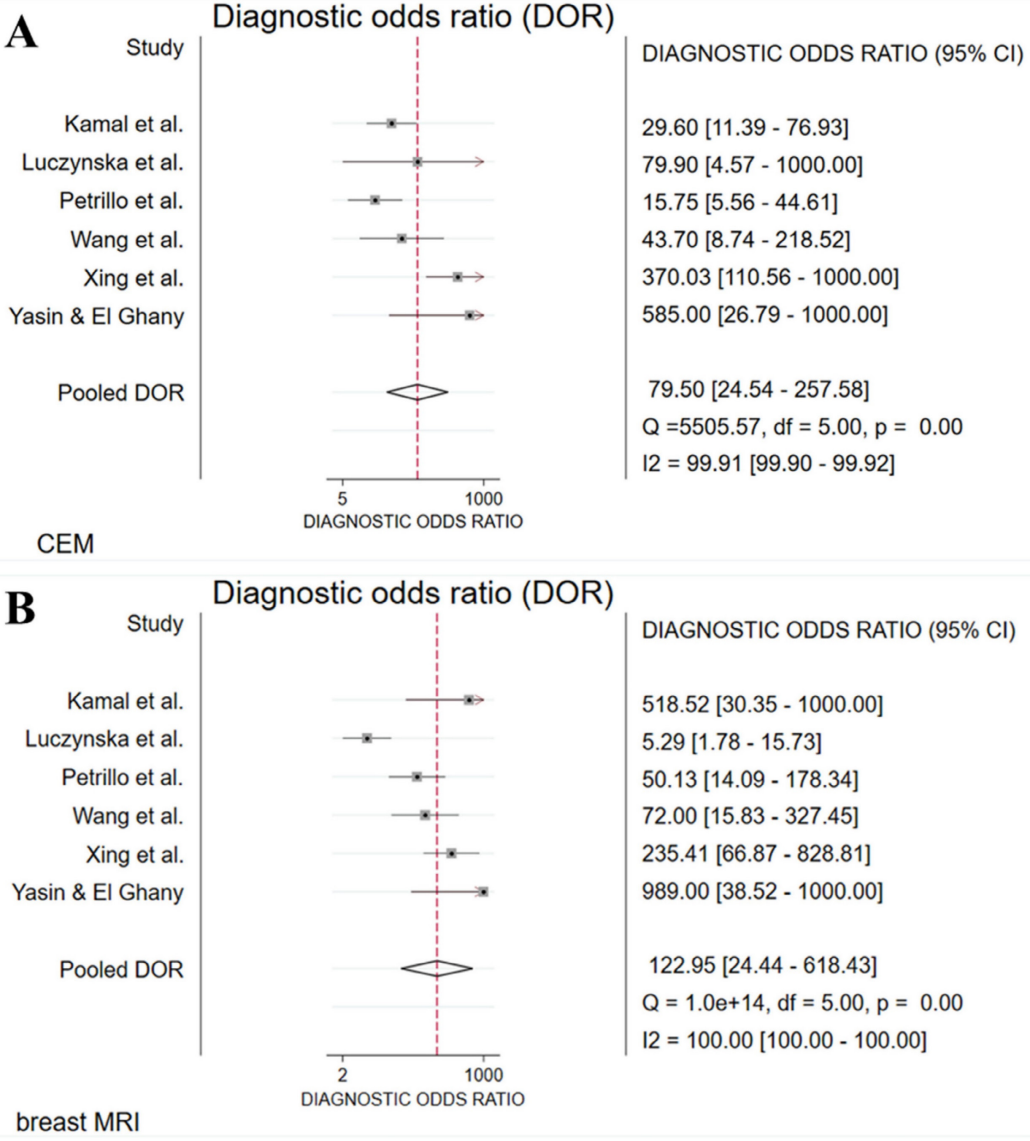
Forest plots of diagnostic odds ratio (DOR) for CEM (A) and breast MRI (B) with pooled values, and I^2^ values.

**Figure 5:**
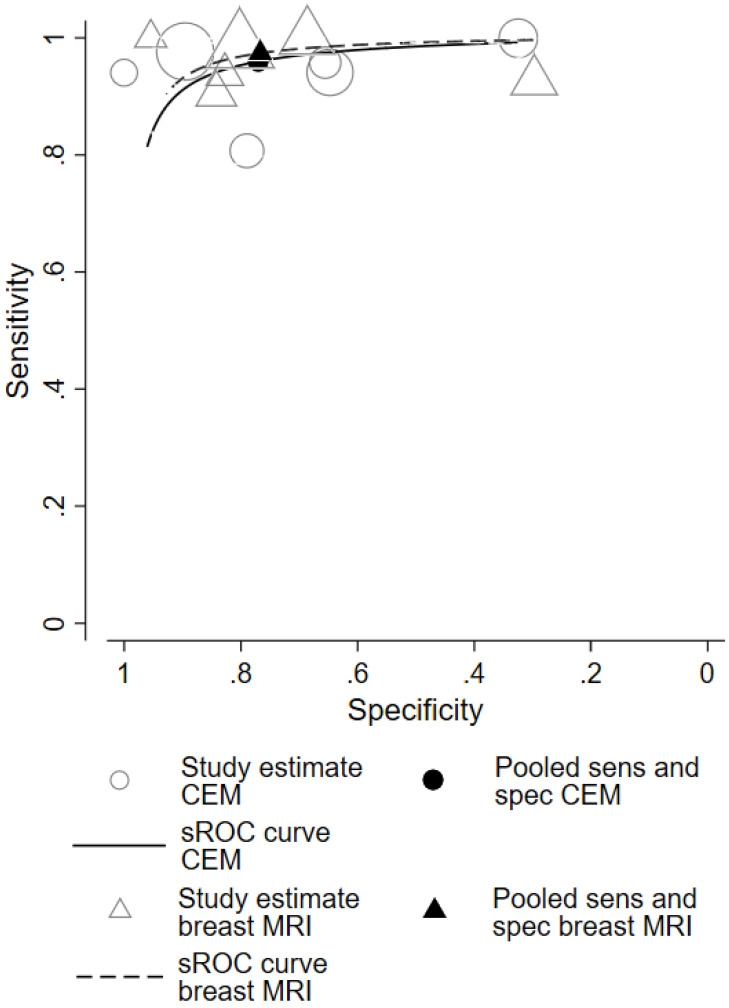
Hierarchical sROC curves of CEM (black line) and breast MRI (black dotted line). The summary points of pooled sensitivity with pooled specificity for CEM and breast MRI are shown with the black circle and black triangle, respectively.

The absolute numbers of TP, FP, FN, TN, and the sensitivity, specificity, positive predictive value, and negative predictive value are given in Table S1. The pooled sensitivity of CEM and breast MRI was 96% (95% CI 90%-99%) and 97% (95% CI 92%-99%), respectively. Pooled specificity was, 77% (95% CI 53%-91%) for CEM and 77% (95% CI 57%-89%) for breast MRI. Figure 2 presents the forest plots for sensitivity and Figure 3 for specificity. Pooled positive likelihood ratio (LR) and pooled negative LR were 4.16 (95% CI 1.86-9.34) and 0.05 (95% CI 0.02-0.13) for CEM and 4.18 (95% CI 2.06-8.48) and 0.03 (95% CI 0.01-0.11) for breast MRI, respectively. The pooled DOR for breast MRI was 122.9 (95% CI 24.4-618.4) versus 79.5 (95% CI 24.5-257.6) for CEM (Figure 4). Between-study heterogeneity was considerable with *I^2^* values exceeding 80%. The hierarchical sROC curves with summary points for CEM and breast MRI are shown in Figure 5. The hierarchical sROC curve is constructed by plotting the sensitivity (true positivity) and false positivity (1 - specificity) of each study.

## Discussion

This systematic review and meta-analysis provide an evidence-based update on the comparative diagnostic performance of CEM and breast MRI in the diagnostic work-up in women with suspicious breast lesions. Pooled sensitivity of breast MRI was slightly higher than that of CEM (97% vs 96%) at similar pooled specificity (77%). The pooled DOR estimates indicate a higher overall diagnostic performance of breast MRI compared to CEM (122.9 vs 79.5). Strict eligibility criteria were applied to ensure that optimal imaging methods were used. Excluded were studies using prototype versions of CEM units and studies from which it could not be deduced with certainty that standard imaging protocols were used. Irrespective of the strict study eligibility criteria applied, the results show that there was still considerable between-study heterogeneity with *I^2^* values higher than 80%. Although studies were performed on CEM systems by two vendors this probably did not contribute to the high *I^2^* values, since recent research showed that diagnostic accuracy of CEM is likely to be vendor system independent 38. One source of the variation between study results could be methodological issues, such as differences in patient populations and variation in reader experience.

Only two meta-analyses have been published that directly compared CEM and breast MRI 23,24. The meta-analysis published in 2019 by Xiang et al. resulted in a pooled sensitivity of 97% for both imaging modalities, but the pooled specificity for CEM (66%) was higher than that for breast MRI (52%) 23. The authors concluded that the diagnostic performance of CEM appears to be more effective than that of breast MRI, because the pooled odds ratio for CEM was 60.15 versus 31.34 for breast MRI. The results of the present meta-analysis do not corroborate these results, because the pooled DOR for breast MRI was much higher (122.9 instead of 31.34) and exceeded the pooled DOR for CEM (79.5). Only two studies of the 13 studies included in the meta-analysis by Xiang et al. were also included in our meta-analysis 23,33,35. The other eleven studies, listed in Supplementary materials 2, were excluded for this meta-analysis for several reasons. Results were untraceable, were published in a conference abstract or proceeding, or in a language other than English. In other studies, CEM was performed on a prototype system or the patient population consisted exclusively of women with an index tumour. The latter studies can answer the question whether CEM and breast MRI differ in the ability to detect additional lesions in the same breast or the contralateral breast 39-41, but cannot provide information on the ability of imaging modalities to discriminate between benign and malignant breast lesions.

The most recent meta-analysis by Pötsch et al. resulted in a lower pooled sensitivity for CEM (91%) than for breast MRI (97%), whereas the pooled specificity for CEM (74%) was higher than that for breast MRI (69%) 24. Overall diagnostic performance of breast MRI (pooled DOR: 73.0) was higher than that of CEM (pooled DOR: 30.4). The authors concluded that there is not yet a conclusive answer on the question as to whether breast MRI is superior to CEM for diagnostic work-up of women in a screening or recall setting. Four of the included studies by Pötsch et al. were also included in current meta-analysis 32,33,35,37. The other three studies were excluded from the current meta-analysis because of the use of a prototype system for low dose CEM 42, a patient population in which all patients were already diagnosed with breast cancer 43, and incorrect absolute numbers of TP, FP, TN and FN 44. In the latter study, Pötsch et al., wrongfully assumed that all 'non-papillomas' were malignant whereas only two non-papillomas were actually malignant. Based on the pooled estimates from the remaining four studies, the difference between sensitivity of breast MRI and CEM becomes much smaller: 98% versus 96% instead of 97% versus 91%, respectively.

The large discrepancies between the results from the three meta-analyses, which are now available, raises concern. Use of different criteria for eligibility of studies resulted in the inclusion of different studies with only partial overlap. To be able to rely on the results of meta-analysis, robust results are needed to guide evidence-based practice. Therefore, we do not agree with the bold statements published earlier by experts Mann and Velthuis 45, which were based on the Pötsch review, stating that CEM would 'take us two steps back in breast imaging' compared to breast MRI. With such claims, we run the risk to prematurely exclude CEM from imaging, when in fact it is a modality which is increasingly being used with much potential 9: more accessible to underserved populations, less expensive, shorter reading times and preferred by patients 4,17,18,46.

Indications for CEM are the same as for breast MRI: preoperative staging 33,36,47 (i.e., tumour size assessment and the detection of multifocal or contralateral breast cancer foci) and response monitoring of patients treated with neo-adjuvant systemic therapy 48-50. We initially aimed to compare the diagnostic performance for these indications as well, but found that the available scientific literature did not provide the necessary data for a systematic review and meta-analysis. For comparison of accuracy of size measurement during pre-operative staging, mean values with standard deviation for size according to histopathology and imaging are needed to calculate summary estimates of mean differences, but these data were generally lacking 33,36,51,52. We identified three studies that compared the performance of CEM and breast MRI in response monitoring, but the use of different definitions of pathological complete response (pCR) hinder comparison of study results 48-50. More research comparing the diagnostic performance of CEM and MRI for these indications is required to draw more robust conclusions in the form of systematic reviews or meta-analyses.

Both this meta-analysis and the other two meta-analyses have limitations. The number of studies which directly compare the diagnostic accuracy of CEM and breast MRI for diagnostic work-up in a recall setting is very limited and the results from individual studies were very heterogeneous. The lack of robustness of the results from the three meta-analyses indicates that the definition of eligibility criteria for inclusion of studies is an important determining factor for the pooled measures of diagnostic accuracy. There is a need for more original diagnostic studies that meet the criteria for valid comparison of the diagnostic accuracy of optimal imaging with CEM and breast MRI using commercially available and generally accepted image acquisition protocols in a well-defined study population of women that are referred for diagnostic work-up.

In conclusion, we showed that sensitivity was high and specificity moderate for both CEM and breast MRI. The higher pooled DOR estimates for breast MRI indicate a higher overall diagnostic performance compared to CEM. Nevertheless, it seems premature to discard CEM as alternative for breast MRI due to the limited number of studies included in this review. Future studies that are directly comparing CEM and breast MRI for various indications are much needed.

## Supplementary Material

Supplementary information, table.

## Figures and Tables

**Table 1:** Quality assessment of the publications using the QUADAS-2 format

Study	Risk of Bias				Applicability concerns		
	Patient Selection	Index Test	Reference Standard	Flow and Timing	Patient Selection	Index Test	Reference Standard
*Kamal et al.* 32	Low	High	High	Low	Low	High	High
*Luczynska et al.* 33	Low	Low	Low	Low	Low	Low	Low
*Petrillo et al.* 34	Low	Low	Low	High	Low	Low	Low
*Wang et al.* 35	Low	High	Low	Low	Low	High	Low
*Xing et al.* 36	Low	Low	Low	Low	Low	Low	Low
*Yasin & El Ghany* 37	High	High	Low	Low	High	High	Low

**Table 2:** Diagnostic accuracy - study characteristics

Author	Kamal et al. 32	Luczynska et al. 33	Petrillo et al. 34	Wang et al. 35	Xing et al. 36	Yasin & El Ghany 37
Year	2020	2015	2020	2016	2019	2019
Study design	P/F	P	P	P	P/F	P
Patients	82	102	70	68	235	50
Age mean ± SD (range)	49.3 ± 10.8 (29-71)	NA	NA	52.9 ± 10.7 (31-82)	51 ± 10 (25-82)	52 (33-83)
Lesions	171	118	90	77	263	56
Disease prevalence lesions	70% [120/171]	69% [81/118]	58% [52/90]	62% [48/77]	67% [177/263]	61% [34/56]
CEM system	GE Senographe Essential	GE SenoBright	Hologic Selenia	GE Senographe DS & GE Senographe Essential	GE Senographe Essential	GE Senographe Essential
CEM contrast	non-ionic contrast agent	Iopromide 370	Visipaque 320	Omnipaque 350	Iohexol 300-350	Visipaque 320
MRI system	1.5T Siemens	1.5T Avanto Siemens	1.5T Magnetom Symphony Siemens	1.5T Signa HDx GE	3.0T Signa HD XT GE	1.5T Magnetom Aera Siemens
MRI contrast	Gd-DTPA (Magnevist)	Gadobutrol (Gadovist)	Gd-DOTA (Magnevist)	Gd-DTPA (Magnevist)	Gd-DTPA (Magnevist)	Gadolinium (not mentioned)

Abbreviations: SD: standard deviation; CEM: contrast-enhanced mammography; MRI: magnetic resonance imaging; P: prospective; F: feasibility; NA: not available.

## Abbreviations

BI-RADS: breast imaging reporting and data system
CEM: contrast-enhanced mammography
breast MRI: contrast-enhanced breast magnetic resonance imaging
CI: confidence interval
DOR: diagnostic odds ratio
FFDM: full-field digital mammography
FN: false negative
FP: false positive
LR: likelihood ratio
NPV: negative predictive value
pCR: pathologic complete response
PPV: positive predictive value
PRISMA-DTA: preferred reporting items for a systematic review and meta-analysis of diagnostic test accuracy
QUADAS-2: quality assessment of diagnostic accuracy studies version 2
Sens: sensitivity
Spec: specificity
sROC: summary receiver operating characteristic
TN: true negative
TP: true positive
