# Supplementary material for: Comparing the Diagnostic Performance of Contrast-Enhanced Mammography and Breast MRI: a Systematic Review and Meta-Analysis
Source: J Cancer. 2023 Jan 1;14(1):174–82. doi: 10.7150/jca.79747 (PMC9809339; doi:10.7150/jca.79747)
Supplement: Supplementary file 1 — Supplementary information, table. [file jcav14p0174s1.pdf]

Supplementary materials

Supplementary materials 1

A list of publications in the database searches that were excluded after data extraction. The publications are listed per reason for exclusion.

Lack of sufficient details on MRI sequence parameters

1. Lee SC, Hovanessian-Larsen L, Stahl D, et al. Accuracy of contrast-enhanced spectral mammography compared with MRI for invasive breast cancers: Prospective study in population of predominantly underrepresented minorities. Clin Imaging. 2021; 80: 364–70.
2. Rudnicki W, Piegza T, Rozum-Liszewska N, et al. The effectiveness of contrast-enhanced spectral mammography and magnetic resonance imaging in dense breasts. Polish J Radiol. 2021; 86: e159–64.
3. Anwar R, Farouk MA, Abdel Hamid WR, et al. Breast cancer in dense breasts: comparative diagnostic merits of contrast-enhanced mammography and diffusion-weighted breast MRI. Egypt J Radiol Nucl Med. 2021; 52: 1–13.

CEM was performed on a prototype system

4. Fallenberg EM, Dromain C, Diekmann F, et al. Contrast-enhanced spectral mammography versus MRI: initial results in the detection of breast cancer and assessment of tumour size. Eur Radiol. 2014; 24: 256–64.
5. Fallenberg EM, Schmitzberger FF, Amer H, et al. Contrast-enhanced spectral mammography vs. mammography and MRI – clinical performance in a multi-reader evaluation. Eur Radiol. 2017; 27: 2752–64.
6. Sumkin JH, Berg WA, Carter GJ, et al. Diagnostic performance of MRI, molecular breast imaging, and contrast-enhanced mammography in women with newly diagnosed breast cancer. Radiology. 2019; 293: 531–40.

Incomplete absolute numbers of TP, FP, FN, and TN or mismatch with research question

7. Bozzini A, Nicosia L, Pruneri G, et al. Clinical performance of contrast-enhanced spectral mammography in pre-surgical evaluation of breast malignant lesions in dense breasts: a single center study. Breast Cancer Res Treat. 2020; 184: 723–31.
8. Carnahan MB, Pockaj B, Pizzitola V, et al. Contrast-enhanced mammography for newly diagnosed breast cancer in women with breast augmentation: Preliminary findings. Am J Roentgenol. 2021; 217: 855–6.
9. Cheung Y-C, Juan Y-H, Lo Y-F, et al. Preoperative assessment of contrast-enhanced spectral mammography of diagnosed breast cancers after sonographic biopsy: correlation to contrast-enhanced magnetic resonance imaging and 5-year postoperative follow-up. Medicine (Baltimore). 2020; 99: e19024.
10. Jochelson MS, Dershaw DD, Sung JS, et al. Bilateral contrast-enhanced dual-energy digital mammography: feasibility and comparison with conventional digital mammography and MR imaging in women with known breast carcinoma. Radiology. 2013; 266: 743–51.
11. Kim EY, Youn I, Lee KH, et al. Diagnostic value of contrast-enhanced digital mammography versus contrast-enhanced magnetic resonance imaging for the preoperative evaluation of breast cancer. J Breast Cancer. 2018; 21: 453–62.
12. Lee-Felker SA, Tekchandani L, Thomas M, et al. Newly diagnosed breast cancer: comparison of contrast-enhanced spectral mammography and breast MR imaging in the evaluation of extent of disease. Radiology. 2017; 285: 389–400.
13. Li L, Roth R, Germaine P, et al. Contrast-enhanced spectral mammography (CESM) versus breast magnetic resonance imaging (MRI): a retrospective comparison in 66 breast lesions. Diagn Interv Imaging. 2017; 98: 113–23.
14. Youn I, Choi SH, Choi YJ, et al. Contrast enhanced digital mammography versus

magnetic resonance imaging for accurate measurement of the size of breast cancer. Br J Radiol. 2019; 92: 20180929.

15. Ferranti FR, Vasselli F, Barba M, et al. Diagnostic accuracy of contrast-enhanced, spectral mammography (CESM) and 3T magnetic resonance compared to full-field digital mammography plus ultrasound in breast lesions: Results of a (pilot) open-label, single-centre prospective study. Cancers (Basel). 2022; 14: 1351.

Supplementary materials 2

A list of included publications in the systematic review and meta-analysis of Xiang et al., which were excluded for current systematic review and meta-analysis. The publications are listed per reason(s) for exclusion.

Publications in languages other than English

1. Jiang TT, Zhang SJ, Li RM, et al. Diagnostic performance of contrast-enhanced spectral mammography. Chin J Radiol 2017; 51: 273–8.
2. Xu HD. A comparative study of contrast-enhanced spectral mammography and magnetic resonance imaging in breast cancer diagnosis. Chin J Gen Pract 2017; 15: 650–3.
3. Yu MQ, Li JC. Comparative study of contrast-enhanced spectral mammography and dynamic contrast- enhanced magnetic resonance imaging in diagnosis of breast cancer. Chin Med Dev 2017; 32: 74–7.
4. Zou M, Wang YJ, Jin B, et al. Comparison of diagnostic efficacy between CESM and CE-MRI in breast diseases. Chin Comput Med Imag 2018; 24: 211–4.

Conference abstract or proceedings

5. Dromain C, Canale S, Bidault F, et al. Value of contrast-enhanced spectral mammography (CESM) in women with newly diagnosed breast cancers compared to MRI: preliminary results. Radiol. Soc. North Am. 2011 Sci. Assem. Annu. Meet., 2011.
6. Li L, Liao L, Germaine P, et al. Abstract P1-02-06: Retrospective comparison of sensitivity and positive predictive value (PPV) of contrast enhanced spectral mammography (CESM) to contrast enhanced breast MRI (BMRI) in 50 malignant

breasts. Cancer Res. 2015; 75: P1-02–6.

Untraceable publication

7. Zhang CZ, Wang QG, Wang JF, et al. Feasibility of contrast-enhanced spectral mammography in the diagnosis of breast cancer. Radiol Pract 2014; 29: 1420–3.

Study in which CEM was performed on a prototype system

8. Fallenberg EM, Schmitzberger FF, Amer H, et al. Contrast-enhanced spectral mammography vs. mammography and MRI – clinical performance in a multi-reader evaluation. Eur Radiol. 2017; 27: 2752–64.

Studies with a patient population consisting exclusively of women with an index tumor

8. Fallenberg EM, Schmitzberger FF, Amer H, et al. Contrast-enhanced spectral mammography vs. mammography and MRI – clinical performance in a multi-reader evaluation. Eur Radiol. 2017; 27: 2752–64.

9. Jochelson MS, Dershaw DD, Sung JS, et al. Bilateral contrast-enhanced dual-energy digital mammography: feasibility and comparison with conventional digital mammography and MR imaging in women with known breast carcinoma. Radiology. 2013; 266: 743–51.

10. Lee-Felker SA, Tekchandani L, Thomas M, et al. Newly diagnosed breast cancer: comparison of contrast-enhanced spectral mammography and breast MR imaging in the evaluation of extent of disease. Radiology. 2017; 285: 389–400.

11. Li L, Roth R, Germaine P, et al. Contrast-enhanced spectral mammography (CESM) versus breast magnetic resonance imaging (MRI): a retrospective comparison in 66 breast lesions. Diagn Interv Imaging. 2017; 98: 113–23.

Table S1: Absolute numbers of true positive, false negative, false positive and true negative lesions and performance.

Author	**# Lesions**	**TP**	**FN**	**FP**	**TN**	**Sensitivity**	**Specificity**	**PPV**	**NPV**
**CEM**									
Kamal et al. [32]	171	113	7	18	33	94% [88-98]	65% [50-78]	86% [79-92]	82% [67-93]
Luczynska et al. [33]	118	81	0	25	12	100% [96-100]	32% [18-50]	76% [67-84]	100% [74-100]
Petrillo et al. [34]	90	42	10	8	30	81% [67-90]	79% [63-90]	84% [71-93]	75% [59-87]
Wang et al .[35]	77	46	2	10	19	96% [86-99]	66% [46-82]	82% [70-91]	90% [70-99]
Xing et al. [36]	263	173	4	9	77	98% [94-99]	90% [81-95]	95% [91-98]	95% [88-99]
Yasin & El Ghany [37]	56	32	2	0	22	94% [80-99]	100% [85-100]	100% [89-100]	92% [73-99]
**Breast MRI**									
Kamal et al. [32]	171	120	0	16	35	100% [97-100]	69% [54-81]	88% [82-93]	100% [90-100]
Luczynska et al. [33]	118	75	6	26	11	93% [85-97]	30% [16-47]	74% [65-82]	65% [38-86]
Petrillo et al. [34]	90	47	5	6	32	90% [79-97]	84% [69-94]	89% [77-96]	86% [71-96]
Wang et al. [35]	77	45	3	5	24	94% [83-99]	83% [64-94]	90% [78-97]	89% [71-98]
Xing et al. [36]	263	174	3	17	69	98% [95-100]	80% [70-88]	91% [86-95]	96% [88-99]
Yasin & El Ghany [37]	56	34	0	1	21	100% [90-100]	95% [77-100]	97% [85-100]	100% [84-100]

Abbreviations: TP: true positive; FN: false negative; FP: false positive; TN: true negative; PPV: positive predictive value; NPV: negative predictive value; CEM: contrast-enhanced mammography; MRI: magnetic resonance imaging.
